# Intra-individual gait patterns across different time-scales as revealed by means of a supervised learning model using kernel-based discriminant regression

**DOI:** 10.1371/journal.pone.0179738

**Published:** 2017-06-15

**Authors:** Fabian Horst, Alexander Eekhoff, Karl M. Newell, Wolfgang I. Schöllhorn

**Affiliations:** 1 Department of Training and Movement Science, Institute of Sport Science, Johannes Gutenberg-University Mainz, Mainz, Rhineland-Palatinate, Germany; 2 Department of Kinesiology, University of Georgia, Athens, Georgia, United States of America; University of Illinois at Urbana-Champaign, UNITED STATES

## Abstract

**Objective:**

Traditionally, gait analysis has been centered on the idea of average behavior and normality. On one hand, clinical diagnoses and therapeutic interventions typically assume that average gait patterns remain constant over time. On the other hand, it is well known that all our movements are accompanied by a certain amount of variability, which does not allow us to make two identical steps. The purpose of this study was to examine changes in the intra-individual gait patterns across different time-scales (i.e., tens-of-mins, tens-of-hours).

**Methods:**

Nine healthy subjects performed 15 gait trials at a self-selected speed on 6 sessions within one day (duration between two subsequent sessions from 10 to 90 mins). For each trial, time-continuous ground reaction forces and lower body joint angles were measured. A supervised learning model using a kernel-based discriminant regression was applied for classifying sessions within individual gait patterns.

**Results and discussion:**

Discernable characteristics of intra-individual gait patterns could be distinguished between repeated sessions by classification rates of 67.8 ± 8.8% and 86.3 ± 7.9% for the six-session-classification of ground reaction forces and lower body joint angles, respectively. Furthermore, the one-on-one-classification showed that increasing classification rates go along with increasing time durations between two sessions and indicate that changes of gait patterns appear at different time-scales.

**Conclusion:**

Discernable characteristics between repeated sessions indicate continuous intrinsic changes in intra-individual gait patterns and suggest a predominant role of deterministic processes in human motor control and learning. Natural changes of gait patterns without any externally induced injury or intervention may reflect continuous adaptations of the motor system over several time-scales. Accordingly, the modelling of walking by means of average gait patterns that are assumed to be near constant over time needs to be reconsidered in the context of these findings, especially towards more individualized and situational diagnoses and therapy.

## Introduction

The ability to walk is a key component of human mobility that is highly related to quality of life. Its assessment enables insight into the system’s behavior, the capacity to identify the severity or nature of a locomotor disease or injury, and to determine the effect of a treatment. Clinical diagnosis and therapeutic interventions are typically oriented on the idea of average behavior and normality. Accordingly, gait patterns are most often modeled using average values of time-discrete variables (e.g. maximum impact force) or average waveforms of time-continuous patterns (e.g. time courses of knee joint angle) from a sample of strides. In consequence, these averaged values are mostly taken as a characteristic representative of a gait stride from an individual or a group. Experimental protocols recording a high number of strides are recommended in order to reduce deviations and provide reliable data that are assumed to be stable over time [1–3].

On a rather coarse view, gait patterns from healthy individuals seem to remain relatively constant over time, even during unconstrained walking. However, a more detailed observation reveals persistent deviations among subsequent executions of a movement task and shows the unlikely possibility of generating identical movement patterns on recurrent efforts of performing the same motor task, even under constant environmental conditions [4–6]. These deviations were traditionally neglected and considered as a maladaptive noise in the system [7] or experimental errors [8–10] that, therefore, need to be minimized during the analysis and treatment of movement. However, understanding the nature of the variability of movement patterns in general has become a major research topic in human movement science [6,11,12].

Movement variability has been identified as an inherent feature of human motor control and learning that occurs naturally throughout multiple levels of movement organization and contributes to deviations in the output of the motor system [5,6,13–17]. Moreover, gait variability is described as a necessary prerequisite in order to ensure an adaptable and flexible locomotion in unpredictable and changing environments [18–20]. Furthermore, gait variability provides information about the maintenance of the health status [21] and can for example be related to age [22–24], disease or injuries [21,25,26], and the risk of falling [27].

The application of concepts and tools from nonlinear dynamics, fractal analysis and chaos theory identified more details about the nature of movement variability and contributed to the understanding that variability is no more considered as equivalent to insignificant noise [7,17,28,29,30]. Movement variability is rather understood to be driven by deterministic and stochastic processes [17]. Accordingly, a normal and healthy gait is characterized by a certain structure and magnitude of variability [18,20,21,31]. White gaussian random noise seems to reflect only a background component in the structure of movement variability [17].

Hence, the modeling of gait patterns by means of averaging several trials that are assumed to be near constant over time (or at least over the duration of a therapeutic intervention) and treating their variability operationally as random deviation within distributional statistics needs to be questioned [17,19,28,32,33]. Whether this modeling is dependent on the usage of time-discrete or time-continuous variables [34] or on the number of considered variables [35] is still pending.

However, a complementary and promising approach towards individualized analysis of gait patterns is provided by the application of more holistic methods in data analysis (e.g., pattern recognition) [34,36]. In this context, the distinction of individual gait patterns [34] and the identification of situational characteristics like emotions [37] or fatigue [38] within intra-individual gait patterns provided further clarification for deterministic features in human movements and thereby indicated advantages of an analysis and treatment of human gait based on individual and situational needs [39,40]. Nevertheless, up to now, it is uncertain how complex deterministic and stochastic processes on multiple levels of movement organization and time-scales affect biomechanical analysis of the individual’s average gait stride patterns or rather the clinical decision-making based on it.

That is, the interaction/influence of natural temporal changes of gait patterns in different time-scales on the evaluation of treatment effect in studies with pre-post design or the monitoring of the rehabilitation process. Although deterministic properties have been shown by means of temporal dependencies in stride-to-stride fluctuations of gait patterns within a single recording or measurement session (intra-session variability) [19,41,42], there is a lack of research of the time-dependent characteristics of gait patterns between repeated measurement sessions (inter-session variability) [43]. In particular, the level of intrinsic persistence of gait patterns has not been well detailed [43]. Previously, the identification of intra-individual changes of gait patterns between days indicated that the intrinsic persistence of gait patterns is smaller than often assumed in gait analysis [40]. Accordingly, we tested the hypothesis that gait patterns as well as their persistence over time are predominantly determined by deterministic processes that lead to time-dependent behavior in terms of natural temporal changes of gait patterns between repeated measurement sessions within a day and different degrees of changes in different time-scales. Therefore, the aim of this study was to examine intrinsic changes in time-continuous gait patterns by: (1) quantifying intra-individual differences in gait patterns between repeated measurement sessions within a day; and (2) quantifying intra-individual differences in gait patterns at different time-scales (i.e., tens-of-mins, and tens-of-hours).

## Methods

### Subjects and ethics statement

Nine physically active subjects (three female, six male; 27.4 ± 3.0 years; 1.74 ± 0.11 m; 73.2 ± 13.3 kg) without gait pathology and free of lower extremity injuries participated in the study. The study was carried out according to the Declaration of Helsinki and all subjects were informed about the experimental protocol and provided their informed written consent. The approval from the ethical committee of the medical association Rhineland-Palatinate in Mainz was received.

### Experimental protocol and data acquisition

The subjects performed 15 gait trials in each of 6 test sessions (S1-S6), while they did not undergo any intervention between the sessions. The time intervals of rest after the first, third and fifth session to the beginning of the subsequent session were 10 mins. The interval between session 2 and 3 and between session 4 and 5 were 30 and 90 mins, respectively.

**Fig 1:**
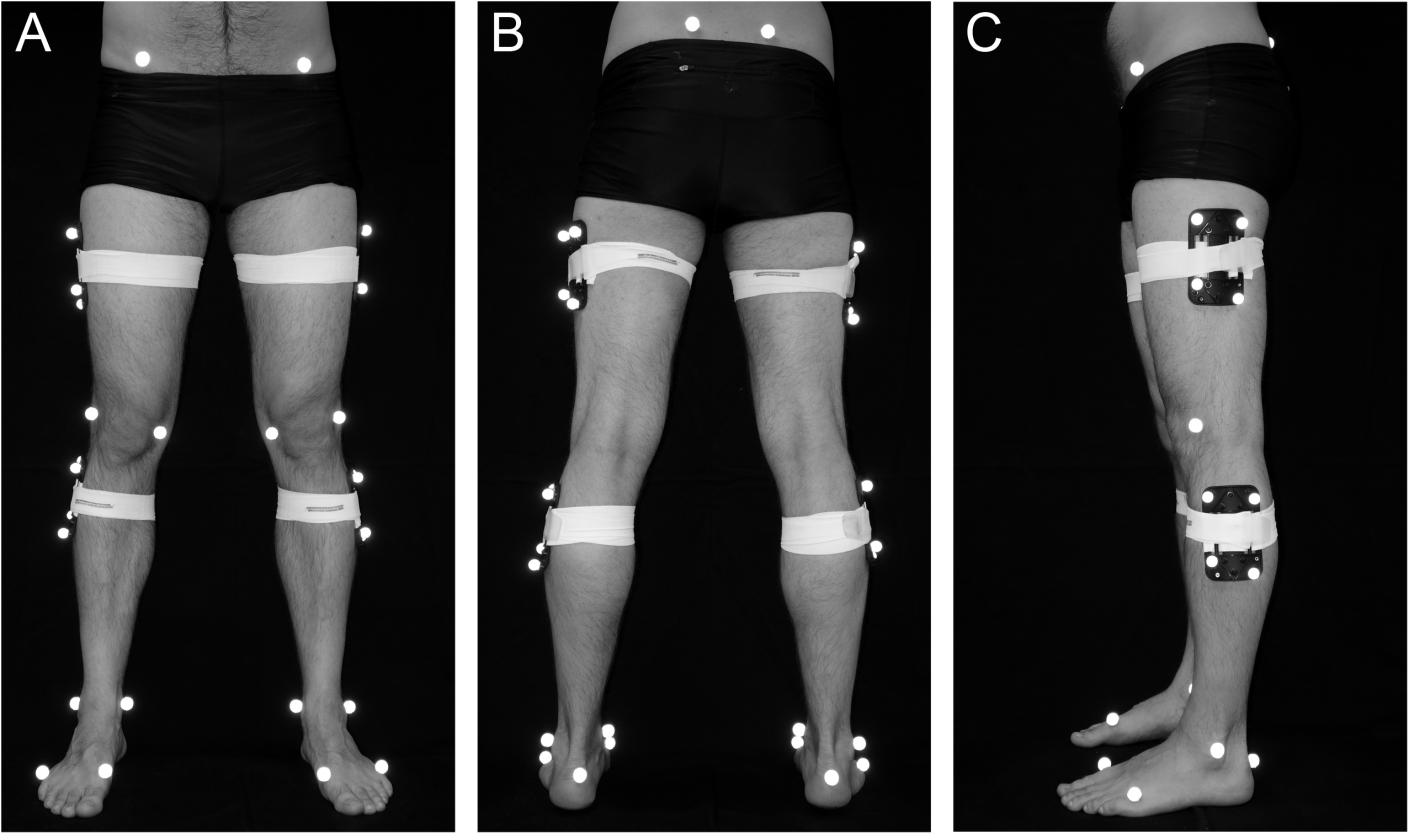
**Lower body marker set in (A) anterior (B) posterior (C) left lateral view.** The markers were placed bilaterally at anterior superior iliac spine, posterior superior iliac spine, femur lateral epicondyle, femur medial epicondyle, fibula apex of lateral malleolus, tibia apex of medial malleolus, posterior surface of calcaneus, head of 1st metatarsus, head of 5th metatarsus and clusters with four markers each at the thigh and shank.

For each trial lower body joint angles as well as ground reaction forces were measured, while the subjects walked on a 10 m path. The subjects were instructed to walk barefoot at a self-selected speed. Kinematic data were recorded using a lower body marker set consisting of 34 retro reflective markers placed on anatomical landmarks (Fig 1). The three-dimensional marker trajectories were captured by nine Oqus 310 infrared cameras (Qualisys AB, Sweden) at a sampling frequency of 250 Hz.

The three-dimensional ground reaction forces were recorded by two Kistler force plates (Type 9287CA) (Kistler, Switzerland) at a frequency of 1000 Hz. The recording was managed time-synchronized by the Qualisys Track Manager 2.7 (Qualisys AB, Sweden). Two experienced assessors attached the markers and conducted the analysis. Every subject was analyzed by the same assessor only. The laboratory environment was kept constant during the investigation.

Before the first session, each subject performed 20 test trials to get accustomed to the experimental setup and to assign a starting point for a walk over the force plates. Before each of the following sessions 5 test trials were performed to consider an effect of practice and control the starting point of the walk. This procedure is described to minimize the impact of targeting on the force plates on the observed gait variables and their variability [44,45]. Additionally, the participants were instructed to watch a neutral symbol (smiley) on the opposing wall of the laboratory to direct their attention away from targeting on the force plates and ensure a natural walk with an upright body position.

### Data processing

The gait analysis was conducted for one gait stride per trial. The stride was defined from right foot heel strike to left foot toe off and was determined using a vertical ground reaction force threshold of 10 N. The computation of the lower body joint angles was conducted by Visual3D Standard v4.86.0 (C-Motion, USA) for hip, knee and ankle in sagittal, transversal and coronal plane. The resulting joint angles were filtered by a second order Butterworth bidirectional low-pass filter at a cut off frequency of 18 Hz. The ground reaction force data were normalized to the body weight measured before every session to exclude the impact of changes in the body mass during the investigation.

Further data processing and analysis was executed by a self-written script within the software Scilab 5.4.1 (Scilab Enterprises, France). Each variable time course was normalized to 100 data points, z-transformed and scaled to a range of -1 to 1 [46]. The z-transformation was executed for kinematic variables for each trial separately and for kinetic variables for all trials. The scaling was carried out in order to prevent numerical difficulties during the calculation of the support vector machines [46] and to ensure an equal contribution of all variables to the classification rates and thereby avoid that variables in greater numeric ranges dominate those in smaller numeric ranges [46]. Scaling is a common procedure for data processing in advance for the classification of gait data [e.g., 34].

### Data analysis

The classification of gait patterns based on joined vectors of all variables, i.e. on input vectors of 1 × 1800 (kinematic) and 1 x 600 (kinetic) per trial. In total, a matrix of size 90 x 1800 (90 = 6 session x 15 trials; 1800 = 100 time points x 2 legs x 3 joints x 3 directions) and 90 x 600 (90 = 6 session x 15 trials; 600 = 100 time points x 2 ground contacts x 3 directions) formed the basis of the classification of one subject for kinematic and kinetic data, respectively. The classification was carried out by supervised learning models using a kernel-based discriminant regression (KBDR) [47] and support vector machines (SVM) [48,49]. Both are supervised learning models for the recognition of patterns and regularities in data. While SVM represent an well-established model for the classification of gait patterns based on joined vectors of time-continuous kinematic and kinetic data [38,40,50,51], KBDR is a recently developed classification approach that has never been applied for the analysis/classification of human movements. While KBDR showed higher classification accuracies than SVM and other models [47], especially on data sets with a small sample size but high dimensions, it seemed to be a promising model for the given classification problem. The ability to distinguish gait patterns of one test session from gait patterns of other test sessions was investigated in a multiclass classification (six-session-classification) and a binary classification for all combinations of two sessions (one-on-one-classification). The multiclass classification used a “one-versus-all” algorithm. The classification rates were conducted for each subject individually by a cross-validation through the leave-one-out-method [52]. The kernel-based discriminant regression was used with a proximal point algorithm and a polynomial kernel function. The degree of the polynomial kernel exp = 0.1, 0.3, …, 3, and proper values for α = 10^−7^, 10^−6^, …, 10^−3^ and β = 0.01, 0.03, …, 10 have been selected using cross validation before training and testing. The L2-regularized L2-loss support vector classification of the Liblinear Toolbox 1.4.1 [53] was used with a linear kernel function. A grid search within the range of the cost parameter C = 2^−5^, 2^−4.75^, …, 2^15^ has been conducted to determine C experimentally before training and testing.

### Statistical analysis

The statistical analysis was conducted using SPSS 21 (IBM, Armonk, New York, USA). The normal distribution of each variable was tested by the test of Shapiro-Wilk. For data that did not significantly deviate from normal distribution, descriptive statistics were presented as means and standard deviations; otherwise, the data were presented as medians and quartile 1-quartile 3. In order to ensure similar walking conditions during the investigation, gait speed, step length, step width and the time from right heel strike to left toe off have been assigned and statistically tested for differences between the six sessions by a repeated measures ANOVA and post-hoc paired t-tests. Compound symmetry, or sphericity, was verified by the test of Mauchly. When the assumption of sphericity was violated, the degrees of freedom were adjusted according to the Greenhouse-Geisser-correction. Likewise, the classification rates for the one-on-one-classification were compared for differences depending on the time duration between two sessions. Therefore, time durations between 2 sessions were grouped into 4 time intervals (T1: 10 mins, T2: 30–50 mins, T3: 90–110 mins and T4: 130–150 mins). A Friedman ANOVA and post-hoc Wilcoxon signed-rank tests were applied when values deviated significantly from normal distribution. The significance levels were set at p = 0.05. In the post-hoc analysis the significance level was adjusted according to the Bonferroni-correction. The effect size eta-square (η^2^) for the repeated measures ANOVA and r effect size for the Wilcoxon signed rank test were calculated and interpreted according to Cohen [54].

## Results

All control variables remained on a similar level during the investigation (Table 1). The repeated measures ANOVA did not show statistically significant differences between the six measurement sessions and confirms comparable walking conditions.

**Table 1:** Mean (standard deviation) of the control variables for each of the six sessions (n = 9).

	session 1	session 2	session 3	session 4	session 5	session 6	repeated measures ANOVA
gait velocity [m/s]	1.50 (0.14)	1.52 (0.14)	1.54 (0.13)	1.55 (0.14)	1.55 (0.15)	1.55 (0.15)	(F _2, 14_ = 1.621; p = .177; η^2^ = .169)
step length [m]	0.77 (0.06)	0.79 (0.06)	0.79 (0.04)	0.79 (0.04)	0.80 (0.05)	0.79 (0.04)	(F _2, 15_ = 1.615; p = .231; η^2^ = .168)
step width [m]	0.13 (0.02)	0.13 (0.02)	0.12 (0.02)	0.12 (0.02)	0.12 (0.02)	0.12 (0.02)	(F _3, 24_ = 2.453; p = .088; η^2^ = .235)
step duration [s]	1.18 (0.08)	1.17 (0.08)	1.15 (0.08)	1.15 (0.08)	1.15 (0.09)	1.15 (0.09)	(F _2, 19_ = 1.846; p = .180; η^2^ = .188)

The six-session-classification resulted in a mean classification rate of 67.8 ± 8.8% (KBDR) and 61.0 ± 9.0% (SVM) for time-continuous ground reaction force curves and 86.3 ± 7.9% (KBDR) and 82.3 ± 8.3% (SVM) for time-continuous joint angle curves. Consequently, the kernel-based discriminant regression was able to classify a mean of 61 (ground reaction force) and 78 (lower body joint angles) out of 90 intra-individual gait patterns correct to the corresponding test sessions.

**Fig 2:**
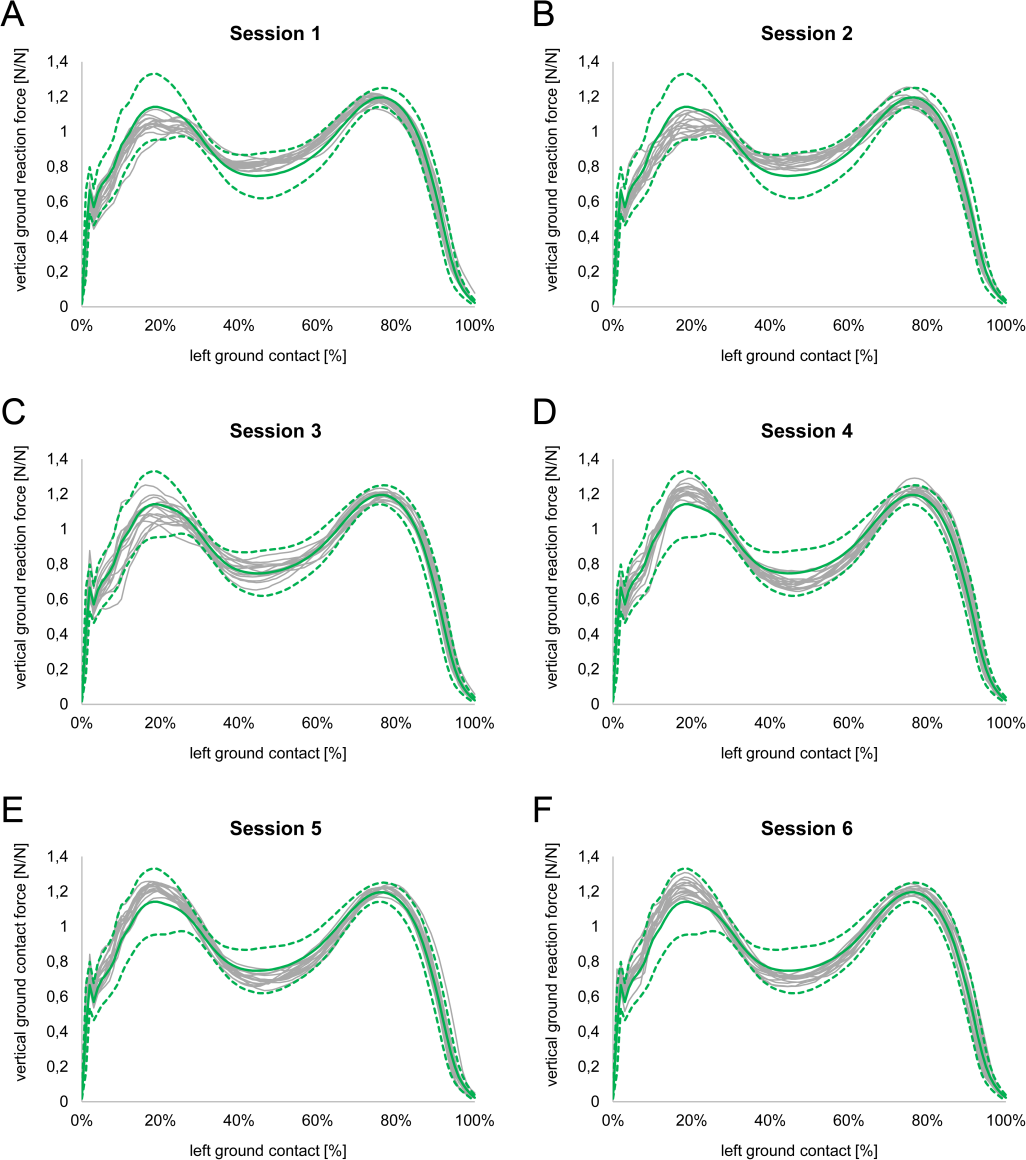
**Vertical ground reaction force of the 15 gait trials (grey) of each of the six sessions (A-F) as well as the global mean (green) and two standard deviations (green dotted) of all 90 gait trials from subject 8 (n = 1)**.

Fig 2 shows the time and amplitude normalized curves of every trial as well as the overall mean curve and curves of enveloping two standard deviations from subject 8. Qualitatively, the curves display different characteristics between the 6 sessions. It is noticeable that the curves did not vary randomly about the global mean curve and rather reveal session specific characteristics (e.g., at ~20% of the gait stride are trials from session 1 & 2 below and trials from session 4, 5 & 6 above the mean curve).

The mean classification rates for the one-on-one-classification disclosed the lowest classification rates for the time duration of ten mins between two test sessions (T1) (Table 2). Furthermore, the results of the one-on-one-classification showed a trend that increasing classification rates go along with increasing time intervals between the sessions.

**Table 2:** Mean ± standard deviation of the classification rates of the one-on-one-classification of kernel-based discriminant regression analysis (KBDR) and support vector machines (SVM) (n = 9).

time interval	sessions	duration	ground reaction force		lower body joint angles	
			KBDR	SVM	KBDR	SVM
**T1 (10 mins)**	S1-S2	10 mins	83.6 ± 12.6%	77.8 ± 14.7%	91.3 ± 6.8%	88.6 ± 7.9%
	S3-S4	10 mins	77.2 ± 15.1%	68.7 ± 17.7%	88.3 ± 5.6%	85.0 ± 6.8%
	S5-S6	10 mins	80.5 ± 8.4%	73.6 ± 6.9%	84.0 ± 10.4%	78.3 ± 10.3%
**T2 (30–50 mins)**	S2-S3	30 mins	79.8 ± 12.5%	73.2 ± 15.3%	93.2 ± 10.5%	90.9 ± 13.3%
	S2-S4	40 mins	93.6 ± 6.1%	88.5 ± 15.5%	99.3 ± 1.5%	98.1 ± 2.9%
	S1-S3	40 mins	92.8 ± 9.3%	88.9 ± 10.5%	98.8 ± 2.5%	97.0 ± 4.3%
	S1-S4	50 mins	95.2 ± 9.8%	93.4 ± 9.5%	99.6 ± 1.1%	99.6 ± 1.1%
**T3 (90–110 mins)**	S4-S5	90 mins	84.5 ± 8.7%	76.9 ± 15.1%	100.0 ± 0.0%	99.8 ± 0.8%
	S4-S6	100 mins	87.3 ± 9.2%	82.6 ± 12.1%	99.6 ± 1.1%	99.3 ± 1.4%
	S3-S5	100 mins	89.9 ± 9.0%	83.3 ± 11.9%	100.0 ± 0.0%	99.6 ± 1.1%
	S3-S6	110 mins	94.8 ± 10.3%	91.4 ± 11.7%	100.0 ± 0.0%	99.8 ± 0.8%
**T4 (130–150 mins)**	S2-S5	130 mins	94.4 ± 5.6%	91.4 ± 8.0%	100.0 ± 0.0%	99.6 ± 1.1%
	S2-S6	140 mins	97.4 ± 4.7%	94.8 ± 6.8%	100.0 ± 0.0%	100.0 ± 0.0%
	S1-S5	140 mins	96.2 ± 6.4%	90.3 ± 11.5%	100.0 ± 0.0%	100.0 ± 0.0%
	S1-S6	150 mins	97.7 ± 3.4%	95.5 ± 5.5%	100.0 ± 0.0%	100.0 ± 0.0%

The median classification rates for the one-on-one-classification based on KBDR for ground reaction force curves resulted in T1 (81.1 (76.1–87.2)%), T2 (90.8 (87.1–97.9)%), T3 (93.3 (85.2–95.0)%) and T4 (97.5 (92.5–100.0)%). The statistical test revealed highly significant results over the four time intervals (Χ^2^ = 19.400; df = 3; p = .000). The pairwise comparisons of T1 and T2 (Z = -2.666; p = .024; r = -.889), T1 and T4 (Z = -2.666; p = .024; r = -.889), T2 and T4 (Z = -2.675; p = .004; r = -.891) as well as T3 and T4 (Z = -2.549; p = .048; r = -.850) showed a statistically significant difference.

The mean classification rates for the one-on-one-classification based on SVM for ground reaction force curves resulted in T1 (73.4 ± 10.2%), T2 (86.0 ± 10.9%), T3 (83.6 ± 10.7%) and T4 (93.0 ± 6.6%). The statistical test revealed highly significant results over the four time intervals (F _3, 24_ = 13.019; p = .000; η^2^ = .619). The pairwise comparisons of T1 and T2 (p = .007) as well as T1 and T4 (p = .001) were statistically significant and T3 and T4 (p = .066) revealed a statistical trend, whereas the other combinations showed no statistically significant difference.

The median classification rates for the one-on-one-classification based on KBDR for joint angle curves resulted in T1 (88.9 (83.7–91.0)%), T2 (100.0 (95.2–100.0)%), T3 (100.0 (100.0–100.0)%) and T4 (100.0 (100.0–100.0)%). The statistical test led to significant differences between the four time intervals (Χ^2^ = 21.409; df = 3; p = .000). The pairwise comparisons were statistically significant for T1 and T2 (Z = -2.547; p = .048; r = -.849), T1 and T3 (Z = -2.666; p = .024; r = -.889) as well as T1 and T4 (Z = -2.666; p = .024; r = -.889), whereas T2, T3 and T4 showed no statistically significant difference.

The median classification rates for the one-on-one-classification based on SVM for joint angle curves resulted in T1 (82.8 (79.2–88.8)%), T2 (98.1 (93.6–100.0)%), T3 (100.0 (99.1–100.0)%) and T4 (100.0 (100.0–100.0)%). The statistical test led to significant differences between the four time intervals (Χ^2^ = 20.68; df = 3; p = .000). The pairwise comparisons were statistically significant for T1 and T2 (Z = -2.547; p = .048; r = -.849), T1 and T3 (Z = -2.666; p = .024; r = -.889) as well as T1 and T4 (Z = -2.668; p = .024; r = -.889), whereas T2, T3 and T4 showed no statistically significant difference.

## Discussion

The present study identified characteristics in intra-individual gait patterns that differ across measurement sessions and revealed permanent, non-random, temporal changes of gait patterns. Discernible changes of time-continuous gait patterns indicate that the gait patterns are not constant over time and their persistence is less than often assumed in gait analysis. Furthermore, the results showed that the amount of randomness within gait patterns is smaller than previously expected [17]. The non-significant differences in basic gait variables like gait velocity, step length and width as well as step duration, imply similar gait conditions and suggest that these variables were not responsible for differences in gait patterns between different sessions. Extrinsic sources of variability in terms of measurement errors were minimized to only a negligible influence on the separation of gait patterns from different sessions. Our findings suggest that inter-session variability is predominantly caused by intrinsic temporal changes of individual gait patterns between measurement sessions [55,56].

The six-session-classification rates of 67.8% (KBDR) and 61.0% (SVM) for time-continuous ground reaction force curves and 86.3% (KBDR) and 82.3% (SVM) for time-continuous joint angle curves differ clearly from a theoretical random classification rate of 16.7% (= chance level for one out of six sessions). This means, both supervised learning approaches for pattern recognition were able to identify characteristics of individual gait patterns that are specific for a certain session and can be used to distinguish gait patterns from other sessions. The distinction of intra-individual gait patterns from repeated measurement sessions indicates continuous changes of gait patterns that appear naturally without any intervention or injury. Accordingly, natural changes of gait patterns can be observed within a single day [40] and the intrinsic persistence of gait patterns is less than often assumed in gait analysis [43]. Supported by previously stated good reliability/repeatability [43], biomechanical diagnoses and therapeutic interventions typically assume that individual gait patterns are near constant without an intervention or injury [1]. Clinical approaches often describe the subject’s gait stride by average values of multiple trials and treat their variability in terms of random deviations within distributional statistics. The identification of temporal changes of gait characteristics point out limitations of those models. The present findings indicate that inter-session variability does not merely feature random characteristics around a stable average curve but rather exhibits incessant temporal changes. Thereby, it is noticeable that the kinematic changes of gait patterns appeared more recognizable or more rapidly than kinetic changes. This might be explained by the fact that the ground reaction forces are determined to a great extent by body mass and gravity, two relatively constant influencing variables. In addition, lower body joint angles exhibit a higher degree of freedom than ground reaction forces and consequently feature a broader range of possible movement solutions. The present findings provide further clarification for deterministic features in human movements, namely time-dependent behavior in terms of natural changes of gait patterns between different test sessions. In accordance with findings from nonlinear measures on the basis of stride-to-stride fluctuations that characterize intra-session variability [7,20,29,41,42], intra-individual changes of gait patterns emphasize a predominant role of deterministic processes in human walking. Consequently, in addition to the identification of information like emotions [37] or the grade of fatigue [38] within intra-individual gait patterns, time-dependent changes provide further clarification for deterministic features in human locomotion.

In addition, the one-on-one-classification shows that increasing classification rates go along with increasing time durations between observations. The lowest, but still high, classification rates of 80.4% (KBDR) and 73.4% (SVM) for ground reaction force curves and 87.9% (KBDR) and 84.0% (SVM) for joint angle curves, respectively, are present for time interval T1 of 10 mins between the sessions. The classification rates of the time intervals T2, T3 and T4 tend to rise sequentially. Increasing classification rates for increasing time intervals between sessions indicate that more and more gait characteristics are changing by time, until gait patterns are completely distinguishable from each other (T4).

However, the results do not provide evidence for a clear linear drift of gait patterns by time but rather indicate changes that appear in different time-scales. Clear differences appear between the classification rates of time interval T1 (tens-of-mins) and T4 (tens-of-hours), whereas the classification rates for the ground reaction force curves are slightly higher for T2 compared to T3. This might be explained by intra-individual differences in adaptations and time-scales of feedback and adaptation processes [33,34,57]. Feedback processes on different levels of movement organization and multiple time-scales may influence the variability of gait patterns within a single measurement session [17,57,58] as well as intrinsic changes between different measurement sessions. Further research is needed in order to examine how deterministic and stochastic processes control stride-to-stride fluctuations as well as intrinsic changes of gait patterns between observations. Moreover, it is interesting how intra- and inter-session variability are connected and if both reflect specific functions of the motor system that may provide different levels of information about human walking.

Apart from this, the identification of natural changes in gait patterns raises many more questions about deterministic processes and time-dependent behavior between observations. For example, do intra-individual gait patterns drift incessantly apart from each other or do they exhibit recurring characteristics in certain time-scales. Which characteristics in intra-individual gait patterns are changing exactly? Are there certain characteristics changing while others remain constant over time? A state-space framework presentation and approaches like the autoregressive integrated moving average analysis might be promising to provide details on these questions.

Changes of intra-individual gait patterns may reflect continuous adaptations of the motor system to so far unknown changes of boundary conditions (e.g. emotions, metabolism, orthostasis) that appear naturally in order to ensure a nearly stable locomotion. The increase of variance in the gait patterns in elderly [23] should be reconsidered with the background of increased anatomical changes with increasing age. The increased gait variability should be considered as a preventative act in order to cope with the bigger anatomical changes caused by aging processes. From this point of view, the rate of intrinsic changes in gait characteristics may as well provide information about the subject. Gait characteristics with more or less constancy could be identified and may provide a basis for an eventual treatment.

In summary, both supervised learning models (KBDR and SVM) lead to comparable results and reinforce the findings. However, the classification accuracy of KBDR was throughout higher than SVM (on average about 5%) and thereby provide first empirical evidence that KBDR seems to be promising for movement analysis, especially for the classification of high-dimensional data based on joined vectors of time-continuous kinematic and kinetic data.

## Conclusion

Intra-individual gait patterns indicate time-dependent characteristics of time continuous gait patterns and a predominant role of deterministic processes in human motor control and learning that have mostly been neglected so far. Natural changes of gait patterns without any externally induced intervention or injury may reflect adaptation processes of the motor system that differ between individuals and appear at different time-scales. Persistent changes of gait patterns seem to be omnipresent in human walking and raise the question if representative temporary gait patterns exist at all. The results emphasize that the modeling of individual gait patterns by means of average patterns that are assumed to be near constant over time needs to be further challenged.

Clinical gait analysis has to be reconsidered in the context of these findings, not only towards more individualized but also towards situational diagnosis, therapy and evaluation of treatment effects. If a system is continuously changing by itself it is difficult to justify repetition oriented therapeutic interventions as a preparation for later events in everyday life [33]. Whether intra-individual changes of gait patterns are incessantly drifting or exhibit recurring characteristics or whether they are a necessary prerequisite for adaptation or both needs further research.

## Supporting information

S1 Table Control variables and classification rates for each participant. (XLSX)
